# The Book World of Medicine and Science

**Published:** 1898-05-07

**Authors:** 


### The Book World of Medicine and Science.

Stammering, Stuttering, and Other Speech Affections. By W. Abbott, M.D., M.R.C.P., formerly Physician to the Hospital for Affections of the Speech, &c. (The Savoy Press: London, 1898. 9th Edition. Pp. 59. Price 1s.)

Stammering and stuttering are affections which cause an infinite amount of misery to individuals, and which conspire to rob the State and learned professions of much talent. It is, in fact, difficult to imagine a career in life which would not be hindered by stammering. It is the more surprising then that so little attention should have been paid to the subject by medical men of repute. With the exception of an admirable lecture by Dr. Colman, we can recall no adequate account of it in contemporary medical literature. Probably—although often aggravated by oral or pharyngeal abnormalities—stammering is, in essence, a failure of certain cortical cells to initiate properly the complex co-ordinations required by polysyllabic speech. Other organs than those of speech may—owing to defective nerve control—"stammer"; witness "stammering bladders" and "stammering respiration." The purpose of Dr. Abbott's pamphlet is sufficiently indicated by his allusion to the gratuitous treatment of a gentleman in reduced circumstances, and the "uniform success which has attended, for twenty years, treatment by correspondence."

A Clinical Text-Book of Surgical Diagnosis and Treatment, for Practitioners and Students of Surgery and Medicine. By J. W. Macdonald, M.D.Edin., Professor of Surgery in Hamline University, Mineapolis. With 328 illustrations. (London: The Rebman Publishing Company, 1898. Price 28s.)

This is a handsome work, well written, well illustrated, and well printed. Moreover, it is a very good practitioner's book, being carefully and systematically arranged, giving what is likely to be useful as a clinical guide, and wisely avoiding pathological discussions. There is nothing about bacteriology, the technique of antiseptics is taken for granted, and elementary studies in regard to bandaging and the application of dressings are passed over as being already understood. The problems to which the book addresses itself are questions which must be answered in regard to every case that presents itself in practice, viz., what is the disease or injury, and what is the proper treatment? This may be a limited field, but it is practical, and we feel sure that the book will be found a very useful one by students and practitioners, especially by the latter, for it is well up to date, and is written in a style which renders reading an easy task. Perhaps if we were inclined to be hypercritical we might say that the author had been rather run away with by his pen, and by the faculty which he evidently possesses for ready writing. Here and there we find expressions which strike one as odd in a sober-minded book on surgery. Speaking of the brain he says, "An organ so essential to the economy, so highly developed, so exquisitely delicate and sensitive, must of necessity be well protected. The skull is the strong casket which contains this precious jewel," &c. Again, "It looks as if the Almighty had traced His own image upon the masterpiece of His handiwork, and recorded the crowning triumph of creation in a language which we are just beginning to learn, and in characters which we hope soon to decipher." We do not pretend to understand what all this means. Yet this is not a mere popular book—it is not that at all—it is a serious work on surgery; but the author's pen runs away with him. He is descriptive and illustrative, and he enjoys it. "When we say that a given area controls a certain motion or a certain part of a limb, we must not assume that the area in question ends abruptly. This is in accordance with Nature's laws. The colours of the rainbow are not sharply defined, but beautifully blended. The light of day does not suddenly cease and the darkness of night begin, but the atmosphere catches the departing rays, and, refracting them to the earth, changes day into night through the mellow light of the gloaming. So it is with the brain: each motion," &c. All this is true no doubt. In fact, we rather like it, but to the English student who loves his Walsham it sounds redundant. It is only occasionally, however, that the author gives way to such flights of fancy. As a general rule the descriptions are good and very clearly expressed. Illustrations are introduced in large numbers, and many of them are admirable, especially some which are reproductions from photographs; amongst them being some terribly weird specimens of disease. We have read this book with interest, and we think that it will be found very useful by any practitioner who wishes to go over his surgery again. There are, moreover, dotted about through its pages many very practical hints as to treatment as well as diagnosis. As we have said, it is well up to date, in illustration of which we may mention that as a final chapter there is a very useful one on the X-rays in surgical diagnosis.

Doctor and Patient: Hints to Both. By Robert Gersuny. Translated by A. S. Levetus, with a Preface by D. J. Leech, M.D., F.R.C.P. (Pp. 78. Price 2s. net. J. Wright and Co., Bristol. 1898.)

This charming essay by Dr. Gersuny admirably contrasts the relations which do exist, and those which should xist, between doctor and patient. Although written in Vienna several years ago, this little book might well be taken as a presentment of the conditions of general practice in London of to-day. What those conditions really are is known only to those who have lived the "laborious days"—and nights—of the general practitioner. The long-suffering, servilitude, and obsequiency required by the middle-class householder of his medical man are enough to crush the soul of all but those who love their work for healing's sake. The relation between doctor and patient is undoubtedly that of a contract. But while the doctor wishes his part of the contract to be that he may do the best for the patient, the patient regards the doctor as a servant, who is to supply "soft soap," the latest thing in tabloids, and certificates for accident insurance whenever required to do so. Increasingly, as Dr. Gersuny says, the family practitioner is becoming a master of ceremonies for the introduction of "specialists," who are called in because "the social position of the patient demands it." The snobbish pleasure taken by the middle classes in the advent of a "specialist" could only be depicted properly by Mr. Gissing. We have had pitiful amusement sometimes at the disappointment shown—even over a mother's death-bed—when the "London doctor" has arrived in "only a cab." It is one of the most pitiful revelations of the meanness of human nature, that in face of the issues of life and death, the man who is to grapple with them should be chosen for the cut of his coat, the colour of his horses, the house he lives in; for anything but his proven skill and professional distinction. Still, as Dr. Gersuny says, "the desire may again rise for doctors who preserve their ideals and who strive to reach a higher goal. Is it necessary that people should only learn at their own cost what they might have foreseen?" "Doctor and Patient" is well written, well translated, and well printed. We wish it could be placed in the hands of all who are "patients."

